# Clinical exploration of marking targeting biopsy in the intraoperative localization value of colon polypectomy

**DOI:** 10.12669/pjms.36.2.756

**Published:** 2020

**Authors:** Ling Yun Song, Qi Lin, Lian Biao Li, Xiu Cheng

**Affiliations:** 1 Dr. Ling Yun Song, M.D. Department of Gastroenterology, Yinzhou No 2. Hospital, Ningbo, Zhejiang, China; 2 Dr. Qi Lin, M.D, Department of Gastroenterology, Yinzhou No 2. Hospital, Ningbo, Zhejiang, China; 3 Dr. Lian-Biao Li, M.D, Department of Gastroenterology, Yinzhou No 2. Hospital, Ningbo, Zhejiang, China; 4 Dr. Xiu Cheng, M.D, Department of Gastroenterology, Yinzhou No 2. Hospital, Ningbo, Zhejiang, China

**Keywords:** Marking targeting biopsy, Polyp of colon, Localization value

## Abstract

**Objective:**

To evaluate the feasibility and safety of marking targeting biopsy (MTB) in the intraoperative localization value of colon polypectomy.

**Methods:**

The clinical data from patients with polyp of colon discovered under colonoscopy from January 2014 to January 2016 were retrospectively analyzed. A total of 87 patients conformed to the inclusion criteria, among them, 43 received colonoscopic polypectomy one week after MTB (MTB group), while 44 underwent colonoscopic polypectomy one week after conventional biopsy (conventional group). The time consumption in colonoscopic treatment, polypectomy rate and postoperative complications between two groups were compared.

**Results:**

The time consumed in operation in the MTB group was 25.5 (±8.6) minutes, while that in conventional group was 42.0 (±20.5) minutes, and the difference was statistically significant (P<0.01). There were a total of 86 polyps in the MTB group, among which 83 were removed, yielding the removal rate of 96.5%. There were altogether 88 polyps in the conventional group, among which 54 were removed, resulting in the removal rate of 61.4%, and the difference was statistically significant (P<0.05). three polyps in the MTB group were detached after MTB, or the wound surface became flat after gross polyp removal, and no polypectomy was required, but the marking targeting solution was clearly visible. two respective polyps in 12 cases in conventional group could not be found in colonoscopic treatment, and 10 of them had respective one polyp that could not be found again. 12 cases in MTB group suffered from abdominal pain after surgery, and no hemorrhage was seen intraoperatively and postoperatively. 10 cases in the conventional group had abdominal pain after surgery, and one case had delayed hemorrhage after surgery. The results between two groups displayed no statistical significance (P>0.05).

**Conclusions:**

The localization value of MTB in colon polypectomy is definitely feasible, safe and effective, which can greatly shorten the time of endoscopic colon polypectomy, mitigate patient sufferings, and reduce the incidence of false negative rate of polyp. It displays favorable clinical application value and is worthy of being promoted in clinic.

## INTRODUCTION

In recent years, colonoscopy becomes increasingly popular. Accordingly, the detection rate of polyp of colon has been greatly improved. Polyp of colon is a common and frequently-occurring disease in colonic mucosa, which can be divided into neoplastic and non-neoplastic. Of them, neoplastic polyp includes tubular adenoma, villous adenoma and mixed adenoma. The canceration rate of adenomatous polyposis coli ranges from 3% to 27%.^1^ Polyps should be removed as early as possible once it is discovered. A variety of endoscopic polypectomy patterns are available with the increasingly improved colonoscopy and operating apparatus. However, polyp localization under colonoscopic polypectomy is difficult due to the site and size of polyp, as well as the special colonic structure. Sometimes, it takes hours to search for the polyp.

## METHODS

Colonoscopy was performed in 87 patients (174 polyps) with colonic polyps from Jan 2014 to Jan 2016 in Yinzhou No 2. Hospital (Ningbo, China), and satisfactory effects were two polyps in different sites, and cancer patients were excluded through pathology. There were 46 males and 41 females, aged 32-78 years (average, 48.46+11.75 years). The patients were randomly divided into two groups, including 43 in the MTB group that received colonoscopic polypectomy 1 week after MTB. There were 22 males and 21 females, with the average age of (44.25+11.02) years. A total of 86 polyps were found, including 4 (4.7%) in the cecum, 9 (10.5%) in the ascending colon, 11 (12.8%) in the transverse colon, 20 (23.2%) in the descending colon, 25 (29%) in the sigmoid colon, and 17 (19.8%) in the rectum. The remaining 44 cases were in the conventional group, who underwent colonoscopic polypectomy 1 week after conventional biopsy. There were 24 males and 20 females, with the mean age of (49.05+10.23) years. A total of 88 polyps were discovered, including 6 (6.8%) in the cecum, 12 (13.6%) in the ascending colon, 16 (18.2%) in the transverse colon, 17 (19.3%) in the descending colon, 27 (30.7%) in the sigmoid colon, and 10 (11.4%) in the rectum. Differences in the general data between two groups were not statistically significant (P>0.05).

**Fig.1:**
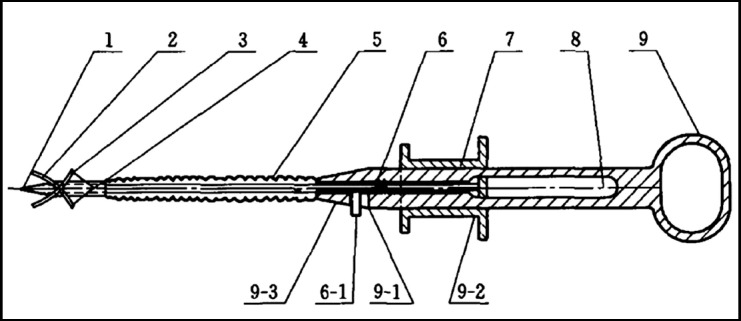
Schematic diagram of the novel endoscopic MTB forceps.

***Notes:*** 1. needle. 2. biopsy forceps. 3. Biopsy forceps fixed socke. 4. Biopsy forceps driving wire. 5. Flexible metal tubing. 6. Infusion tube. 7. Control block of entry needle and biopsy forceps. 8. Infusion tube adapting piece.9. Pull rod handle.

The electronic colonoscope Olympus 290 Electronic Endoscopic System, ERBE Electrotome System, marking targeting biopsy forceps (Fig.1) and the matched 1:10 medical Indian ink stain, disposable entry needle, disposable snare, noradrenaline, 0.9% sodium chloride injection, and Nanjing minimally-invasive harmony clip were used.

Patients with colonoscopy indications were enrolled, and those with biopsy contraindications, such as taking aspirin tablet or clopidogrel tablet within the past 3 days, were excluded. The patients were informed in detail about the procedure preoperatively, and had signed the informed consent for endoscopy and MTB. Conventional bowel preparation was conducted in all patients, 30ml simethicone emulsion was administered 2-4 hour prior to surgery to remove the bubbles, and conventional blood pressure measuring and cardio-pulmonary function evaluation were performed. For patients in MTB group, the MTB forceps were first inserted into the biopsy channel during operation; subsequently, the marking targeting solution was connected, the target was determined, the biopsy forceps were stretched, and the needle was penetrated into the mucosa. The marking targeting solution was pushed until the slight bulging of mucosa was pimple-like, suggesting effective injection. The handle was pulled back to close the biopsy forceps after injection marking targeting, which marked the completion of marking targeting and biopsy, and each injection volume was about 0.1-0.3 ml. During the examination, marking targeting in the polyps basal side and small biopsy were recommended, and a second marking targeting in the peri-polyp mucosa was required for cases with unclear marking targeting or marking targeting solution loss after biopsy. The marking targeting points should be timely washed with purified water after completion of injection in all marking targeting points, and any unclear point should be supplemented with marking targeting immediately. Patients with errhysis after MTB would be washed with iced 0.9% sodium chloride injection or 1:10000 noradrenaline solution, and observed for five minutes to ensure no active bleeding. The wound surface should be clipped using the Nanjing minimally-invasive harmony clip for patients with massive bleeding or hematoma.

### Post-MTB treatment

Patients were informed of the notices after MTB, postoperative fasting for 2-4 h was conducted, and slag-free liquid or semi-liquid was given within 3 days after surgery. The patients were advised to rest appropriately and avoid strenuous exercise. Patients with clear pathology and without cancer were admitted one week later, and a second bowel preparation was conducted for endoscopic polypectomy after completing related examinations.

### Colonoscopic Polypectomy

The colonoscope was inserted in all patients to the cecum, and any polyp discovered was given submucosal injection using the disposable entry needle and 0.9% sodium chloride injection. After sufficient polyp elevation, the disposable snare was used for the loop ligature of polyp base, the polyp was then resected under the 300W electrocoagulation plus electrosurgical excision model, and the wound surface was closed using one or two harmony clips.

### Postoperative Treatment

The patients were allowed liquid diet after six hour of fasting, and they were advised to rest in bed and avoid strenuous exercise. Besides, the patients were given intravenous injection of 80 mg carbazochrome sodium sulfonate for hemostasis for once a day for three days continuously. The stool routine was re-examined after surgery, and patients with negative occult blood result could be discharged. For patients with abdominal pain after surgery, the abdomen plain film in standing position was performed to exclude perforation.

### Observation Indexes

The procedure time in polypectomy and number of the removed polyps in both groups were recorded, and the polypectomy rates were compared. Meanwhile, intraoperative and postoperative adverse reactions were observed. Statistically significant differences in the time consumption in colonoscopic treatment, polypectomy rate and adverse reaction of polypectomy between two groups were examined.

### Statistical Methods

The SPSS 13.0 software was adopted for statistical analysis. Measurement data were expressed as mean± standard deviation and analyzed using t test. Enumeration data were analyzed using chi-square test. A difference of P<0.05 was deemed as statistically significant.

## RESULTS

Among the 43 patients receiving MTB, 36 (83.7%) were successful in a single marking targeting. seven patients were successful after two or above marking targeting, resulting in the overall response rate of 100% (43/43). Among patients receiving two and above marking targeting, five had the colonic polyps of <10 mm in diameter, two suffered from marking targeting solution outflow after MTB, and the mark was not obvious after washing, so a second marking targeting was conducted in peri-polyp mucosa.

### Polypectomy

The operation time in 43 patients of MTB group was 25.5 (±8.6) minutes, while that in conventional group was 40.2 (±28.5) min, and the difference between two groups was statistically significant (*t*=4.34, *P*<0.01). 83 polyps were removed in the MTB group, with the removal rate of 96.5%; whereas 54 polyps were removed in the conventional group, resulting in the removal rate of 61.4%, and the difference between two groups was statistically significant (χ^2^=3.847, *P*<0.05). The polyp diameter in MTB group ranged from 5 to 15 mm, with an average of 13 (±2.5) mm, that in conventional group was 5-16 mm, with an average of 11 (±1.5) mm, and no statistically significant difference was discovered (*t*=1.765, *P*>0.05). 3 polyps in the MTB group were detached after MTB, or the wound surface became flat after gross polyp removal, and no polypectomy was required, but the marking targeting solution was clearly visible.1 respective polyp in 10 cases could not be found again, and they were advised to re-examine colonoscopy done year later.

### Adverse Reactions

There were a total of 174 polyps in 87 patients, 12 cases in MTB group had abdominal pain after polypectomy, and no bleeding was observed intraoperatively and postoperatively. Ten cases in conventional group had abdominal pain after polypectomy, and one had delayed bleeding after polypectomy, the abdominal pain was relieved on the day after polypectomy, but the differences between two groups were not statistically significant (*P*>0.05). one patient had bright red bloody stools (about 10 ml each time) for twice on the second day after surgery, As a result, the patient was given intravenous injection of Agkistrodon snake venom hemocoagulase (2 u), and the bleeding ceased on that day. No chief complaints such as perforation in and after MTB and polypectomy were reported in other patients.

## DISCUSSION

Polyp of colon is the most common disease in digestive tract polyp and can occur in all sites of the colon. This requires that we should be cautious during colonoscopy, and polypectomy should be carried out as early as possible for the discovered polyp of colon.^2^

Currently, the endoscopic polypectomy methods include biopsy removal, endoscopic mucosal resection (EMR), and endoscopic submucosal dissection (ESD), and each method has its own merits and demerits. Biopsy removal is generally adopted for polyp < 5 mm in diameter, while EMR is frequently employed for polyp 5-20 mm in diameter. Polyp >20 mm, nylon rope combined with titanium clip or ESD can be adopted to remove the polyp. There are numerous plicae in the intestinal tract, which together with intestinal gurgling and the cleaning degree of preoperative bowel preparation will directly affect the time consumption of endoscopic polypectomy and the time to search for polyp. When localizing polyp through the distance from the anus, the endoscope body is not in the straightening status due to the influence of stool residue and operational skill. As a result, such localization method can hardly be effective. Repeated endoscope advance or withdrawal will stimulate the intestinal tract, which leads to aggravated intestinal gurgling, adding to the difficulty in searching for the polyp.^3^ Therefore, the authors have adopted the intestinal mucosa MTB technique.

The MTB technique uses the tailor-made MTB forceps for biopsy in the meantime of staining marking in the same site, which is convenient for re-examination recognition.^4^ Entry needle is previously used as the body cavity mucosal marking target, but the marking target and biopsy site can hardly be localized at the same point; therefore, its accuracy is also doubted. In MTB, biopsy and marking targeting are concentrated in the specific biopsy forceps. The marking targeting machine is disposable, which can prevent cross infection and plantation. Besides, it has short operation time, which can greatly mitigate patient sufferings. The applied marking targeting solution (Indian ink) is one of the most extensively used medical stains.

According to the study from Karoui^5^, the marking existence time ranged from 5 to 126 weeks after marking in the stomach using MTB forceps. Furthermore, the marking tended to be stable after 52 to 130 weeks and could be maintained for a long time. The overall response rates at 26, 52 and 78 weeks of follow-up were 96.43%, 90.48% and 90.48, respectively. The marking existence rates in all sites within the stomach were also compared, which was the highest in the lesser curvature of stomach. Hosono^6^ had carried out MTB to monitor and evaluate the effect in 65 chronic atrophic gastritis patients, and their results indicated favorable effect. Chiu^7^ had performed MTB in 72 patients to monitor and evaluate the marking targeting effect in esophageal mucosa, and the single marking targeting successful rate was 100%. The overall response rates after 6, 12, 16 and 20 months of follow-up were 100%, 91.3%, 88.7% and 85.5%, respectively.

The intestinal mucosa has thin intestinal wall, loose submucosal tissue, and more abundant submucosal vessels and lymphatic vessels, the elevation time is short after submucosal injection, the submucosal liquid is likely to diffuse.^8^,^9^ Therefore, MTB injection is linked with greater risks of intestinal wall penetration and submucosal hematoma. Additionally, when carrying out MTB in patients with small polyp, the marked mucosa may be potentially removed along with the polyp in biopsy, which may affect the successful rate of initial marking targeting.^10^ However, satisfactory effect can still be attained after increase in marking targeting frequency and deepening of marking targeting depth.

## CONCLUSION

This study reveals that MTB can be used for clinical outcome monitoring and effect evaluation of intestinal mucosal disease. In this study, 22 patients developed transient mild upper abdominal pain and another three had mild errhysis after biopsy, but no other serious adverse reactions are found. Besides, MTB has greatly shortened the endoscopic treatment time, reduced the false negative rate of polyp, alleviate patient sufferings, and largely enhance the patient compliance in colonoscopy follow-up. These findings reveal that MTB is safe and reliable in colonic polyp localization removal, which is worthy of being promoted in clinic.

### Authors Contributions:

**LYS, QL:** Conceived, designed and editing of manuscript, is responsible for integrity of research. **LBL, XC:** Did data collection and manuscript writing **LYS:** Did review and final approval of manuscript.
